# Locally Advanced Cervical Cancer: Multiparametric MRI in Gynecologic Oncology and Precision Medicine

**DOI:** 10.3390/diagnostics15222858

**Published:** 2025-11-12

**Authors:** Sara Boemi, Matilde Pavan, Roberta Siena, Carla Lo Giudice, Alessia Pagana, Marco Marzio Panella, Maria Teresa Bruno

**Affiliations:** 1 Department of Radiology, Fondazione IRCCS Cà Granda, University of Milan, 20122 Milano, Italy; 2 Department of General Surgery and Medical-Surgical Specialties, Gynecological Clinic, University of Catania, 95123 Catania, Italymt.bruno@unict.it (M.T.B.)

**Keywords:** cervical cancer, multiparametric MRI, staging, parametrium, PET/CT, image-guided adaptive brachytherapy (IGABT), diffusion-weighted imaging (DWI), radiomics, artificial intelligence

## Abstract

**Background:** Locally advanced cervical cancer (LACC) represents a significant challenge in oncology, requiring accurate assessment of local extent and metastatic spread. Multiparametric magnetic resonance imaging (mpMRI) has assumed a central role in the loco-regional characterization of the tumor due to its high soft-tissue resolution and the ability to integrate functional information. **Objectives:** In this narrative review, we explore the use of mpMRI in the diagnosis, staging, and treatment response of LACC, comparing its performance with that of PET/CT, which remains complementary for remote staging. The potential of whole-body magnetic resonance imaging (WB-MRI) and hybrid PET/MRI techniques is also analyzed, as well as the emerging applications of radiomics and artificial intelligence. The paper also discusses technical limitations, interpretative variability, and the importance of protocol standardization. The goal is to provide an updated and translational summary of imaging in LACC, with implications for clinical practice and future research. **Methods:** Prospective and retrospective studies, systematic reviews, and meta-analyses on adult patients with cervical cancer were included. **Results:** Fifty-two studies were included. MRI demonstrated a sensitivity and specificity greater than 80% for parametrial and bladder invasion, but limited sensitivity (45–60%) for lymph node disease, lower than PET/CT. Multiparametric MRI was useful in early prediction of response to chemotherapy and radiotherapy and in distinguishing residual disease from fibrosis. The integration of MRI into Image-Guided Adaptive Brachytherapy (IGABT) resulted in improved oncological outcomes and reduced toxicity. The applications of radiomics and AI demonstrated enormous potential in predicting therapeutic response and lymph node status in the MRI study, but multicenter validation is still needed. **Conclusions:** MRI is the cornerstone of the local–regional staging of advanced cervical cancer; it has become an essential and crucial tool in treatment planning. Its use, combined with PET/CT for lymph node assessment and metastatic disease staging, is now the standard of care. Future prospects include the use of whole-body MRI and the development of predictive models based on radiomics and artificial intelligence.

## 1. Introduction

Cervical cancer remains a leading cause of oncological morbidity and mortality in women globally. According to the most recent data from the Global Cancer Observatory (GLOBOCAN 2022), it is the fourth most common cancer in women, with approximately 600,000 new diagnoses and over 340,000 deaths each year [1]. Cervical cancer is strongly associated with HPV infection and represents the final stage of a natural history that begins with CIN3, the most significant precursor lesion [2]. The introduction of cytological screening programs and, more recently, vaccination against human papillomavirus (HPV) has led to a significant reduction in incidence in high-income countries. Conversely, in low- and middle-income regions, cervical cancer remains a leading cause of oncological death in women [3].

From a clinical point of view, cervical cancer shows a wide spectrum of presentation, ranging from early cancer, often diagnosed thanks to pap-smear, HPV testing and colposcopy, to locally advanced neoplasms (locally advanced cervical cancer, LACC), which in developing countries represent approximately 40% of new diagnoses [4]. The management of LACC is particularly complex and requires an accurate definition of the extent of the disease, both at the loco-regional level (cervix, parametrium, vagina, pelvic wall, contiguous organs) and at the systemic level (lymph node involvement and distant metastases). The biological understanding of HPV-related carcinogenesis provides the basis for interpreting the morphological and functional changes detectable on MRI during tumor assessment.

Historically, cervical cancer staging was clinical, based on a gynecological examination and supplemented by transvaginal ultrasound, cystoscopy, and proctoscopy. The 2018 revision of the FIGO classification represented a turning point, introducing the possibility of including imaging and pathological data in staging [5]. In the current scenario, magnetic resonance imaging (MRI) is considered the modality of choice for locoregional assessment of cervical cancer, due to its high-resolution soft tissue visualization and ability to integrate morphological and functional information. Meanwhile, 18F-FDG PET/CT is regarded as the preferred technique for evaluating lymph node involvement and distant metastases [6,7].

The main sequences used by MRI include T2-weighted, T1-weighted, diffusion-weighted imaging (DWI) with apparent diffusion coefficient (ADC) map and dynamic contrast-enhanced study (DCE-MRI).

### 1.1. T2-Weighted Sequences

T2 sequences are considered the backbone of cervical imaging. The high contrast between the cervical stroma, typically hypointense, and the tumor tissue, which appears hyperintense, allows for precise delineation of the lesion. Thanks to multiplanar planes (axial, sagittal, coronal), T2-weighted MRI allows for: defining tumor margins and their possible infiltration into the cervix or vagina; assessing extension toward the pelvic wall or adjacent organs; measuring the size of the mass with high accuracy, a crucial parameter for FIGO staging.

This sequence is also essential for planning imaging-guided brachytherapy, as it allows for precise delineation of target volumes (HR-CTV, IR-CTV).

### 1.2. T1-Weighted Sequences

Although less specific for defining the stroma, T1-weighted sequences have a complementary role: they identify the presence of intralesional hemorrhage (T1 hyperintensity); they recognize necrotic areas, which appear hypointense; they allow the evaluation of bone marrow infiltration or bone metastases in combination with contrast.

Another aspect concerns the ability to distinguish mucinous lesions or rare variants that may present spontaneous T1 hyperintensity due to protein content.

### 1.3. Diffusion-Weighted Imaging (DWI) and ADC Map

Diffusion-weighted sequences are the primary functional tool of MRI. Cervical cancer, being a highly cellular tumor, shows marked diffusion restriction with hyperintensity on high-b DWI images and corresponding hypointensity on ADC maps.

ADC (Apparent Diffusion Coefficient) values have assumed clinical relevance because they correlate with: biological aggressiveness: lesions with lower ADCs tend to have a worse prognosis; therapeutic response: early increases in ADC during chemoradiation are associated with favorable outcomes; potential prognostic role: several radiomic studies have demonstrated that intralesional heterogeneity of diffusion indices predicts a higher risk of recurrence [8].

### 1.4. Dynamic Contrast-Enhanced MRI (DCE-MRI)

Dynamic sequences with contrast medium (gadolinium) allow the study of tumor vascularization and neoangiogenesis [9]. Cervical cancer typically exhibits early, intense, and inhomogeneous enhancement, distinct from the stroma. Dynamic analysis allows us to characterize areas of increased angiogenic activity, which correlate with aggressiveness; distinguish residual tumor from post-treatment fibrosis; provide quantitative parameters (time to peak, wash-in slope, wash-out slope) that can be used in predictive radiomics and AI models.

In summary, the combination of different MRI sequences allows the integration of morphological (T2, T1) and functional (DWI/ADC, DCE) information, making multiparametric MRI a key tool for the staging, prognosis and therapeutic monitoring of ACC.

### 1.5. Clinical Monitoring of the ACC

At the same time, PET/CT provides greater sensitivity in detecting lymph node metastases, especially small ones, and distant disease. MRI, however, remains superior in terms of anatomical and topographical definition and in assessing relationships with pelvic structures, making the two methods highly complementary [10].

Another important aspect is MRI’s ability to differentiate the main histological subtypes of cervical cancer, which present distinct radiological and biological characteristics.

• Squamous cell carcinoma (70–75%): appears as a solid mass hyperintense on T2, often with spiculated margins and marked diffusion restriction on DWI; on DCE, it shows early and irregular enhancement [11].
• Endocervical adenocarcinoma (20–25%): characterized by endophytic growth along the canal, it may appear as glandular thickening with mucinous hyperintense areas on T2 or T1 (due to protein content); Diffusion restriction is generally less marked. MRI is essential for assessing extension towards the endometrium and lower uterine segment [12].
• Adenosquamous and rare variants: present heterogeneous characteristics, with mixed signals on T2 and inhomogeneous enhancement [11].
• Neuroendocrine carcinomas (<5%): highly aggressive tumors, often small but highly cellular, with extremely low ADC and intense early enhancement, associated with a poor prognosis [13].
• Rare mucinous variants (e.g., adenoma malignum, signet ring cell carcinoma): may appear hyperintense on T1 due to mucinous accumulation and cystic on T2, sometimes simulating benign lesions [14].

These characteristics demonstrate that MRI is not only a staging tool, but also a method capable of providing indirect prognostic information related to tumor biology. Radiomic studies have also highlighted that quantitative parameters extracted from images, such as ADC values and intralesional heterogeneity, are associated with poor prognosis and a higher risk of recurrence [15,16].

MRI has also acquired a crucial role in assessing treatment response: volumetric reduction in T2 and early increase in ADC values during chemoradiation correlate with improved outcomes. Furthermore, it has proven irreplaceable in imaging-guided adaptive brachytherapy (IGABT), allowing for precise delineation of target volumes (HR-CTV and IR-CTV) and contributing to improved local control and reduced toxicity, as demonstrated by the EMBRACE-I trial [17].

In recent years, the integration of radiomics and artificial intelligence (AI) in MRI image analysis has opened new perspectives. The extraction of complex quantitative parameters and the application of machine learning-based predictive models have shown promising capabilities in predicting therapeutic response and risk stratification [18]. However, the lack of standardization and multicenter validation currently limits its routine clinical applicability.

In summary, multiparametric MRI is now a cornerstone method in the staging and management of LACC, with a role that goes well beyond simple anatomical assessment, including prognostic, therapeutic and personalized medicine aspects.

### 1.6. Technical Limitations, Artifacts, and Inter-Observer Variability

Despite the high accuracy reported in the literature, advanced imaging in locally advanced cervical cancer still presents some technical limitations that can affect its clinical reliability. In particular, mpMRI is susceptible to motion artifacts related to intestinal peristalsis, respiration, and the presence of gas, which can interfere with the quality of sequences, particularly DWI. Additional causes of image degradation include the presence of prosthetic materials, surgical clips, or intrauterine devices, which can alter the assessment of pelvic structures. Even in PET/CT, physiological FDG uptake in the intestine, bladder, or uterus can generate false positives, especially in the early staging phase.

Another critical issue is interobserver variability, particularly in the assessment of parametrial and lymph node invasion. Several studies have highlighted how operator experience significantly influences the interpretation of T2-weighted and DWI sequences, with a risk of overestimating or underestimating tumor extension. This variability can have significant implications for staging and treatment planning, especially in the absence of reading by radiologists experienced in gynecologic imaging.

These observations underscore the need for greater specific training, as well as the implementation of diagnostic centralization strategies in referral centers, where possible. In this context, the development of artificial intelligence-based algorithms aimed at automatic segmentation and standardization of interpretation could also be a useful tool in reducing subjective variability.

Compared to previous reviews, the added value of this work lies in its multiparametric approach specifically focused on LACC. In particular, it provides a comparative analysis of mpMRI and PET/CT across the main decision-making phases—diagnosis, lymph node and distant staging, and treatment response assessment. Moreover, it explores in depth the emerging roles of WB-MRI and PET/MRI from a translational perspective, while integrating insights from radiomics and artificial intelligence as applied to gynecologic oncology.

The objective is not merely to summarize the existing evidence, but to offer a critical and clinically oriented perspective that reflects the ongoing technological evolution of diagnostic imaging in this field. The focus on LACC, which still represents a significant proportion of new diagnoses worldwide and is associated with a less favorable prognosis, justifies the need for an updated and targeted summary that can serve as a useful tool for radiologists, oncologists, and radiation oncologists in their daily clinical practice.

## 2. Materials and Methods

This review was conducted with a structured narrative approach, with the aim of providing a critical synthesis of the available literature on the role of multiparametric magnetic resonance imaging (mpMRI) in the diagnosis, staging, and management of locally advanced cervical cancer (LACC). The search strategy was inspired by the PRISMA guidelines to ensure transparency and methodological rigor in study selection, but it was not designed as a systematic review or meta-analysis.

### 2.1. Sources and Search Strategy

A search was conducted on PubMed/MEDLINE, Embase, and Scopus, covering the period from January 2000 to April 2025. The choice of this time period was motivated by the introduction and progressive diffusion of functional MRI sequences (diffusion and perfusion), which have significantly changed the diagnostic and prognostic approach to this tumor. Combinations of MeSH terms and keywords were used, including cervical cancer, locally advanced cervical cancer, magnetic resonance imaging, multiparametric MRI, diffusion-weighted imaging, dynamic contrast-enhanced MRI, PET/CT, brachytherapy, radiomics, and artificial intelligence. Aggregate quantitative data analysis (e.g., forest plots, combined AUCs) or systematic risk of bias assessment using tools such as QUADAS-2 was not performed, as the focus of this study is the critical integration of clinical and technical evidence to support medical practice. The reported sensitivity and specificity data were selected as representative examples and presented in qualitative comparison.

### 2.2. Inclusion and Exclusion Criteria

Only English-language articles on patients with locally advanced cervical cancer (FIGO stages IIB-IVA) that reported data on the diagnostic accuracy of imaging techniques (MRI, PET/CT, ultrasound); prognostic or predictive correlations (imaging biomarkers); therapeutic applications (adaptive brachytherapy); and innovative perspectives (radiomics, artificial intelligence) were included. The following were excluded: case reports, studies with small caseloads (<10 patients); works not specifically related to locally advanced cervical cancer (early stages or recurrences); studies on animal models; articles not available in English or with incomplete data; and duplicate contributions.

### 2.3. Selection Process

Article selection was carried out independently by two authors (initials SB and MP), while any disagreements were resolved collaboratively. Relevant data were manually extracted and organized according to main themes (diagnosis, staging, therapeutic response, emerging techniques). A systematic risk of bias assessment was not performed, as this was not part of the narrative review design, but the methodological quality of the included studies was taken into account in the critical discussion of the results.

The literature analyzed includes several levels of evidence: primarily meta-analyses and systematic reviews (Woo et al., 2018 [19]; Chen et al., 2024 [20]; Olthof et al., 2024 [21]), which provided aggregate data on the accuracy of mpMRI in locoregional staging and its comparison with PET/CT. Prospective studies (e.g., Zhu et al., 2021 [22]; Pálsdóttir et al., 2021 [23]; Jeong S et al., 2024 [24]) evaluating MRI performance in real-world clinical settings were also included, as were retrospective studies and case series (RetroEMBRACE, Sturdza et al., 2016 [25]; Wilson et al., 2021 [26]; Dickie et al., 2017 [27]) with a focus on functional parameters and quantitative biomarkers. Particularly noteworthy were prospective multicenter trials, such as EMBRACE-I (Pötter et al., 2021 [17]), which established the role of MRI in adaptive brachytherapy planning, representing the highest level of evidence available on the topic.

Furthermore, international consensus documents and guidelines (ACR 2025; ESGO/ESTRO/ESP 2024 [28]) were considered, confirming the pivotal role of mpMRI in staging according to the FIGO 2018 classification and its integration with PET/CT in lymph node assessment. Finally, more recent studies on radiomics and artificial intelligence were analyzed (Reinhold et al., 2020 [16]; Liu et al., 2024 [29]; Jeong et al., 2024 [24]; Cai et al., 2024 [18]), which, despite methodological and validation limitations, open innovative perspectives towards precision medicine.

In summary, the review integrates consolidated evidence from high-quality methodological studies with emerging and innovative contributions, offering a broad and critical overview of the state of the art and future prospects of imaging in locally advanced cervical cancer.

### 2.4. Organization of Results

In total, approximately 52 studies (Supplementary Table S1).

The extracted data were summarized in the results section and subsequently critically interpreted.

### 2.5. Standardization of Imaging Protocols

One of the main challenges to the uniform clinical use of advanced imaging techniques in locally advanced cervical cancer is the absence of standardized acquisition and interpretation protocols. Currently, there is considerable inter-center variability in technical parameters—such as slice thickness, coil type, functional sequences, DWI parameters, and b-values in mpMRI—as well as in the diagnostic criteria for parametrial invasion, therapeutic response, and lymph node involvement.

This heterogeneity limits reproducibility and comparability of results across studies and can also hinder communication between radiologists, gynecologic oncologists, and radiation oncologists.

To address these issues, the adoption of internationally shared guidelines defining minimum recommended protocols for mpMRI, PET/CT, and hybrid techniques—tailored to disease stage and clinical timing (diagnosis, restaging, follow-up)—appears essential. Experience from systems such as PI-RADS for the prostate and LI-RADS for the liver demonstrates that standardization can enhance diagnostic accuracy, improve training, facilitate research, and support the integration of automated and radiomic approaches.

In the selected studies, mpMRI protocols showed some variability, but common technical features can still be identified. Typically, T2-weighted images were obtained in three orthogonal planes (axial, sagittal, coronal) with 3–4 mm slice thickness, 0–1 mm interslice spacing, and acquisition matrices ranging from 256 × 256 to 320 × 320. DWI sequences were generally acquired in the sagittal or axial plane with b-values between 0 and 800 s/mm^2^, often accompanied by ADC maps for functional evaluation.

Dynamic contrast-enhanced MRI (DCE-MRI) was usually performed in the axial plane with rapid sequential acquisitions after intravenous gadolinium injection, useful for assessing neoangiogenesis and treatment response. In some cases, isotropic 3D sequences were also reported, allowing multiplanar reconstruction.

Despite residual inter-center differences, these parameters reflect a progressive trend toward harmonization of MRI protocols for locally advanced cervical cancer, as recommended by international societies such as ESUR and ESGAR.

## 3. Results

**Figure 1:**
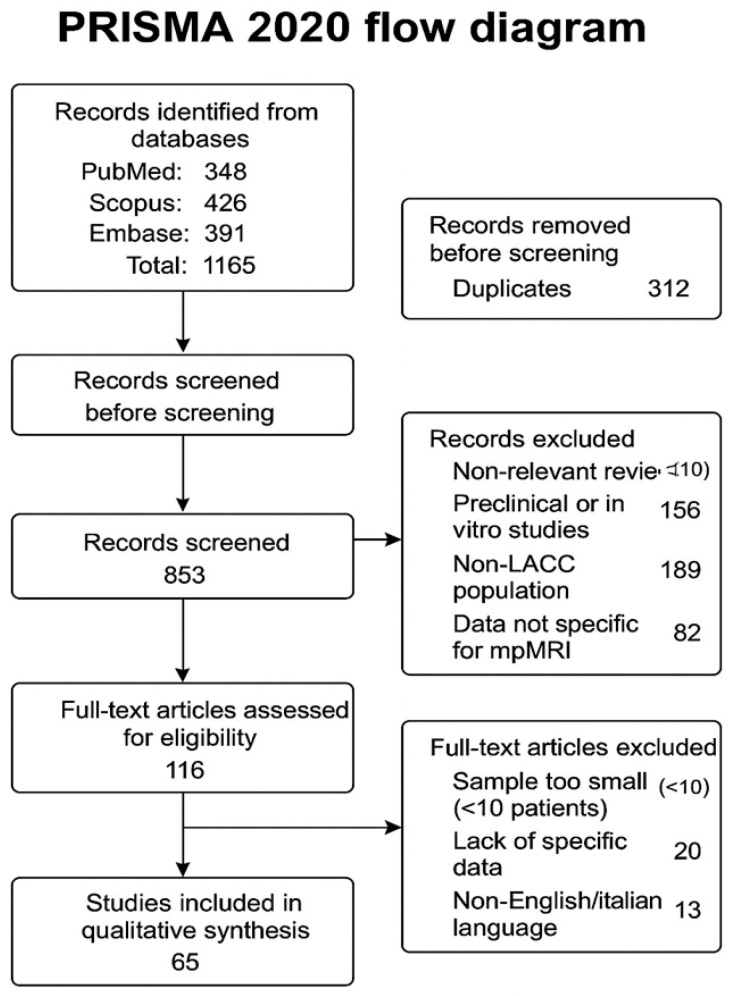
Flow Diagram—Study selection (n = 65 included); based on PRISMA Diagram model.

The initial search identified 1165 potentially relevant articles. After removing duplicates (312), 853 articles remained for screening of titles and abstracts. Subsequently, 18 studies with small caseloads, 189 works not relevant to LACC and 13 articles not available in English were excluded. At the end of the process, a total of 65 studies were included (meta-analyses, prospective and retrospective studies, multicenter trials, guidelines and contributions on biomarkers and radiomics) (Figure 1 flow diagram adapted from the PRISMA model).

Studies identified by database search:

PubMed: 348.

Scopus: 426.

Embase: 391.

Total identified: 1165.

• Duplicates removed: 312.
• Screened studies (title and abstract): 853.
• Studies excluded after screening: 737.
  ○ Main reasons:
    1. Inappropriate review (n = 310).
    2. Preclinical or in vitro studies (n = 156).
    3. Non-LACC population (n = 189).
    4. Data not specific to mpMRI (n = 82).
• Articles assessed in full text: 116.
• Excluded after full text: 51.
  ○ Main reasons:
    1. Too small a sample size (<10 patients) (n = 18).
    2. Lack of specific data (n = 20).
    3. Language other than English/Italian (n = 13).
• Studies included in the final qualitative synthesis: 65.

To ensure greater adherence to clinical practice, the collected data were presented according to the stages of the FIGO 2018 classification (Table 1), analyzing for each parameter the accuracy of multiparametric magnetic resonance imaging and other imaging techniques in order to ensure consistency with clinical practice and promote an integrated vision of diagnostic performance, but also of therapeutic implications and future development prospects (Table 2).

### 3.1. Stage IB3–Bulky Tumors (>4 cm)

Stage IB3 according to the 2018 FIGO classification includes cervical tumors larger than 4 cm but confined to the cervix, thus without visible extracervical extension. This subgroup, defined as “bulky tumor”, is particularly clinically relevant due to its worse prognosis compared to stages IB1 and IB2 and because it significantly impacts therapeutic strategy.

In this context, magnetic resonance imaging (MRI) remains the imaging method of choice for accurate tumor volumetric assessment, surpassing both bimanual clinical assessment and ultrasound, thanks to its excellent soft-tissue resolution and the ability to obtain precise measurements in three orthogonal planes.

T2-weighted sequences are considered the most accurate method for three-dimensional tumor measurement, clearly surpassing clinical examination and transvaginal ultrasound. Bulky tumors appear as expansive masses with intermediate-to-high signal, replacing the normal cervical architecture. The lesion often appears heterogeneous, with central necrotic areas associated with greater biological aggressiveness [17].

T1-weighted sequences, however, generally show iso- or hypointense signal, but may reveal hyperintensity in cases of intratumoral hemorrhage or the presence of mucin, both common features of large lesions [18].

Diffusion-weighted imaging (DWI) sequences and ADC mapping are essential for assessing tumor cellularity. Bulky tumors show marked diffusion restriction, with significantly reduced ADC values, proportional to the high cellular density typical of these lesions [30,31]. The use of dynamic contrast-enhanced MRI (DCE-MRI) also allows analysis of the tumor’s vascular pattern: inhomogeneous or peripheral enhancement is often observed, with central avascular areas corresponding to necrotic zones [17].

Clinically, the presence of a bulky tumor is considered an unfavorable prognostic factor. Sizes > 4 cm are associated with an increased risk of lymph node metastases, local recurrence, and hematogenous dissemination. For this reason, in many centers, radical surgery is avoided in favor of combined treatment with concurrent chemoradiation (CRT), followed by high- or low-dose brachytherapy [19].

**Figure 2:**
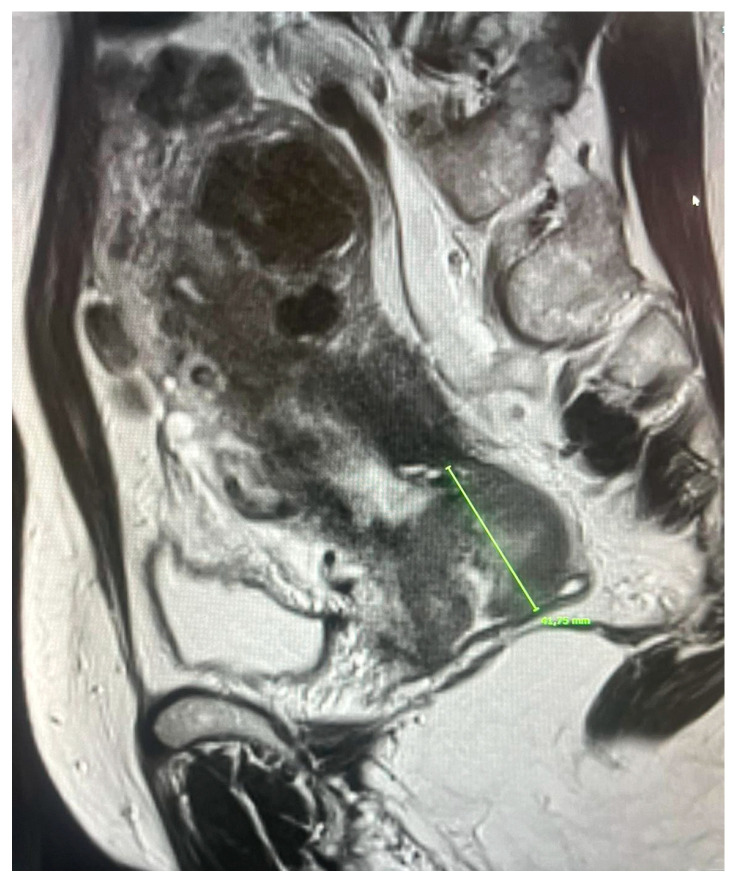
Wide-field sagittal section through the pelvis with measured lesion.

This approach has demonstrated greater efficacy in local disease control and functional preservation of pelvic organs. In this context, MRI is also crucial for the planning of adaptive brachytherapy (IGABT), allowing the precise delineation of target volumes (HR-CTV) and reducing the risk of underdosing, as demonstrated by prospective multicenter studies (EMBRACE-I) [17] (Figure 2).

### 3.2. Stage IIB–Parametrial Invasion

Parametrial invasion is a key element of the 2018 FIGO classification, as it marks the transition from a stage that is still surgically treatable to a condition requiring CRT as the standard of care. Accurate parametrial assessment therefore has crucial clinical implications.

The traditionally used bimanual clinical assessment is characterized by limited sensitivity and specificity and is highly operator-dependent [31]. In this context, multiparametric magnetic resonance imaging (mpMRI) has become the gold standard.

Numerous studies have demonstrated the accuracy of MRI in assessing parametrial invasion. The meta-analysis by Woo et al. (2018), which included over 1600 patients, reported a pooled sensitivity of 76% and a specificity of 94%, confirming the method’s high ability to exclude invasion [19]. A more recent systematic review by Chen et al. (2024) [20] confirmed sensitivity values between 66% and 78% and specificity >90% in the different cohorts analyzed, highlighting how the combination of T2-weighted sequences with DWI and DCE further increases diagnostic performance.

From a radiological perspective, the most reliable signs identified in the literature include discontinuity of the hypointense band of the cervical stroma, interruption of the parametrial fat layer, and signal asymmetry. Studies emphasized that loss of stromal line continuity on T2 represents the most accurate marker of parametrial extension, while Rockall and Hricak highlighted the usefulness of DCE in increasing specificity, especially in equivocal cases due to inflammatory processes or post-biopsy fibrosis [30,31,32,33].

In addition to morphological sequences, functional sequences have provided a significant contribution. Clinical studies have shown that DWI-derived ADC increases early in response to therapy, and that higher values predict the absence of residual parametrial invasion [34].

In parallel, DCE-derived perfusion parameters, such as K^trans^ and extracellular volume fraction, have been associated with less biological aggressiveness and better outcomes [35].

However, the literature also highlights important limitations: the sensitivity of MRI in identifying small parametrial infiltrations remains modest, and interobserver variability has been reported to be significant, particularly among less experienced radiologists. For this reason, the ESGO/ESTRO/ESP guidelines (2024) recommend MRI as the first-line method for assessing parametrial invasion, but emphasize the need for standardized protocols and operator training [36].

In summary, mpMRI has proven superior to clinical assessment in the study of parametrial extension, with high specificity and negative predictive value, representing the most reliable tool for identifying patients who can be managed with chemo-radiotherapy rather than radical surgery.

### 3.3. Stage IIIA–Extension to the Lower Third of the Vagina

In the 2018 FIGO classification, lower vaginal third involvement defines stage IIIA and leads to a worsening prognosis, as well as a notable change in treatment planning. Correct identification of this condition is therefore crucial to avoid staging errors and appropriately plan treatment.

Traditional clinical examination and transvaginal ultrasound have shown limitations in terms of sensitivity and specificity, especially in cases of bulky tumors or in the presence of bleeding. Multiparametric MRI, thanks to the tissue contrast of T2-weighted sequences, has proven superior. Under normal conditions, the vaginal wall appears as a thin, hypointense line on T2, separating the mucosa from the surrounding environment. Its loss or interruption is the key sign of tumor infiltration [30,31]. However, this finding can be distorted by inflammatory processes or motion artifacts, which is why integration with functional sequences is now considered essential.

Prospective studies have confirmed the accuracy of this method. Pálsdóttir et al. (2021) reported a specificity of 96% and a higher overall accuracy compared to transvaginal ultrasound (96% vs. 83%) [23]. Similarly, Zhu et al. (2021) showed that MRI achieves a specificity of 92%, compared to 80% for ultrasound, with a significant advantage in tumors that diffusely infiltrate the vaginal wall [22].

Other methods have also been compared with MRI. Vaginosonography, studied by Vidal Urbinati et al. (2022), showed a sensitivity and specificity of around 89%, demonstrating a fair degree of accuracy especially in small tumors [37]; however, MRI maintained an advantage in bulky cases, where ultrasound assessment is more complex.

The literature agrees that mpMRI is the reference technique for assessing vaginal extension, both for its ability to distinguish lower third involvement (crucial for staging) and for its ability to precisely define the length and volume of the disease. This information is essential not only for staging purposes but also for brachytherapy planning, where accurate delineation of the vaginal extension allows for more precise design of target volumes. Clinically, involvement of the lower third of the vagina leads to caudal extension of external beam radiation fields, potentially impacting late-stage morbidity, particularly sexual functioning and quality of life. Accurate assessment of vaginal extension is therefore essential not only for correct staging but also for adequate treatment planning.

Functional sequences may further contribute. Specifically, DWI has been proposed to increase sensitivity in cases where T2 assessment is uncertain, while DCE may provide clues to infiltration based on an early mucosal enhancement pattern. These parameters, although not yet fully validated on a large scale, could contribute to further improving diagnostic accuracy in the future.

**Figure 3:**
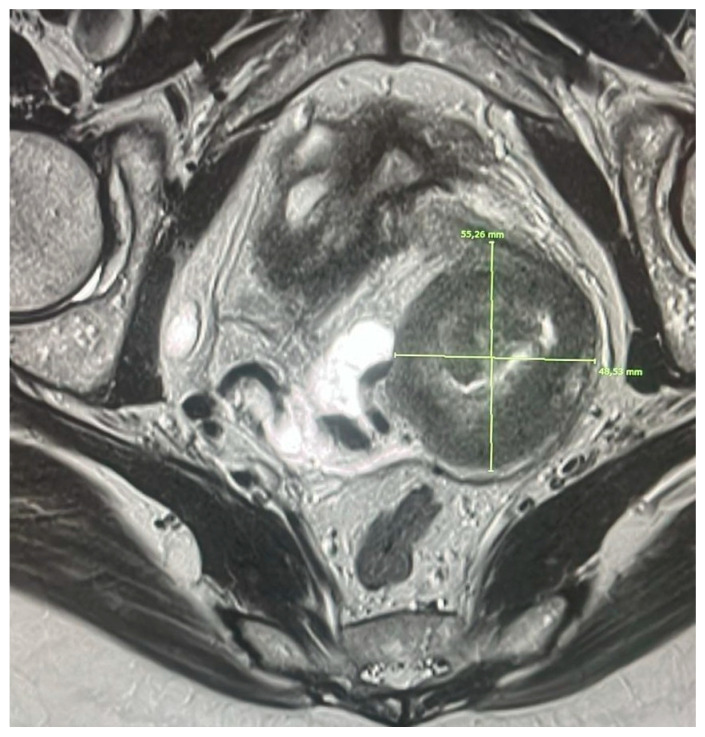
Axial T2 image, small fov with lesion. The lesion infiltrates the vaginal phonics and determines inhomogeneity of the parameters bilaterally as per infiltration.

In conclusion, MRI represents the most reliable method for assessing vaginal extension, with superior performance compared to transvaginal ultrasound and vaginosonography, and with a direct impact on correct staging and treatment planning (Figure 3).

### 3.4. Stage IIIB–Extension to the Pelvic Wall and/or Hydronephrosis

According to the 2018 FIGO classification, stage IIIB is defined by the presence of tumor extension to the pelvic wall or the appearance of hydronephrosis secondary to ureteral obstruction. Both of these parameters are of significant prognostic importance, as they identify patients with more advanced disease, less surgical treatment options, and the need for an integrated radiochemotherapy approach.

Clinical assessment using bimanual examination is unreliable for identifying pelvic wall involvement, especially in cases of bulky tumors or those with limited accessibility. Multiparametric MRI, particularly T2-weighted sequences, represents the gold standard for this assessment. When the lesion reaches or exceeds 3 mm from the muscular wall or directly involves the iliac vessels, the disease is considered to have extended to the pelvic wall. This type of extension represents a key indicator of locally advanced and inoperable disease.

Bourgioti et al. (2016), in a prospective study, reported an overall accuracy of 92–95% in the diagnosis of pelvic wall extension, emphasizing the importance of loss of the fat layer between the tumor and the internal obturator muscles and direct proximity to the iliac vessels as predictive signs of invasion [38]. Chen et al. (2024) confirmed these observations, indicating a sensitivity of between 70% and 80% and a specificity of over 90% in the cases analyzed [20].

DWI and DCE sequences can increase sensitivity in cases where morphological findings are not conclusive. In particular, DWI allows for the detection of areas of diffusion restriction in the pelvic wall, while DCE helps distinguish between simple contact and true infiltration, based on an early enhancement pattern [30,31].

The presence of hydronephrosis, also a criterion for stage IIIB, is easily identified on MRI as dilation of the renal collecting system. On MRI, it manifests as hyperintense dilation of the ureteropelvic system on T2-weighted sequences, while any signs of blood stasis or urinoma may be visible on T1 as hyperintense areas. Again, MRI allows for the distinction between obstruction due to tumor compression and other causes of urinary tract alterations. The clinical importance of hydronephrosis is far from negligible: according to a study published by Garza-Montúfar et al. (2024), its presence was found to be an independent negative prognostic factor, significantly associated with a reduction in overall survival, particularly in cases where timely urinary diversion is not performed [39].

From a therapeutic standpoint, stage IIIB excludes surgery and requires treatment based on concomitant chemoradiation (CRT). In patients with bilateral hydronephrosis or impaired renal function, preventive urinary diversion—using ureteral stents or percutaneous nephrostomies—is often necessary before initiating cancer therapy. This multidisciplinary approach, which combines advanced imaging and urological supportive interventions, is the cornerstone of the integrated management of advanced cervical cancer [40].

### 3.5. Stage IIIC–Lymph Node Involvement

The 2018 FIGO revision introduced stage IIIC to indicate the presence of lymph node metastases, whether pelvic (IIIC1) or para-aortic (IIIC2), thus recognizing the crucial prognostic role of lymph node involvement. Survival of patients with lymph node involvement is significantly reduced, regardless of regional extent, and correct identification of this condition directly impacts radiotherapy planning, particularly the size of radiation fields.

MRI has demonstrated significant limitations in lymph node assessment, as morphological criteria (size > 10 mm on short-axis view, irregular contour, loss of the fatty hilum) do not allow for the identification of micrometastases or metastases in normal-sized lymph nodes [31]. Studies reported by Özen, Adam and Choi have shown a sensitivity ranging from 45% to 60% and a specificity between 82% and 93%, values that reduce the reliability of the method in this setting [10,41,42].

In comparison, 18F-FDG PET/CT has demonstrated superior diagnostic performance, thanks to the ability to detect neoplastic metabolic activity even in normal-sized lymph nodes. A prospective multicenter study published by Olthof et al. (2024) [21] directly compared MRI, CT, and PET/CT in the detection of lymph node metastases in patients with locally advanced cervical cancer. The results showed a sensitivity of 74% and a specificity of 93% for PET/CT, significantly higher than those of MRI, establishing it as the preferred imaging modality in lymph node staging [21,43]. In particular, for para-aortic lymph nodes, PET/CT reaches sensitivities of 72–100% and specificities up to 96%, compared to lower values for MRI (sensitivity 42–66%) [44,45]. These data are particularly relevant because the presence of metastatic PALNs substantially changes the scope of radiotherapy and therapeutic strategies. The evidence collected, however, is not homogeneous: some studies highlight wide variations in accuracy values, attributable to differences in imaging protocols, reading criteria, and the population analyzed. Furthermore, direct correlation with histopathological data is often absent or limited, weakening the strength of the conclusions.

In recent years, there has been a growing interest in radiomics and artificial intelligence as complementary tools in lymph node characterization. Liu et al. (2024) demonstrated that radiomic models built on T2-weighted and DWI sequences can predict the presence of lymph node metastases with an AUC > 0.80, outperforming morphological assessment alone [29]. Similarly, Jeong et al. (2024) [24] developed deep learning algorithms capable of achieving accuracies > 85% in predicting therapeutic response and lymph node status. However, the lack of multicenter validation and standardized protocols still limits their clinical application [24].

Clinically, documentation of lymph node metastases requires classification in IIIC and the inclusion of positive lymph nodes in radiotherapy fields, which intensifies treatment and may impact toxicity. Integration of MRI and PET/CT is therefore essential in current practice: MRI provides precise anatomical definition and volumetric planning, while PET/CT offers superior sensitivity for detecting metastatic disease. Combined, they enable a more accurate and individualized therapeutic strategy.

Despite these limitations, MRI maintains a crucial role, especially in defining pelvic anatomy, assessing topographic relationships between lymph nodes and adjacent structures, and integrating morpho functional data into radiotherapy planning. In particular, the information provided by MRI is crucial in personalizing treatment fields in conformal radiotherapy and adaptive brachytherapy, contributing to precision medicine even in the absence of metabolic confirmation by PET/CT [40].

Recently, whole-body MRI with DWI sequences (DWIBS) has been proposed as a radiation-free alternative with potential for comprehensive assessment. The studies by Sun et al. and Tanaka et al. documented good performance of WB-MRI, especially for bone metastases, suggesting a possible complementarity with PET/CT [46,47].

### 3.6. Stage IVA–Bladder or Rectal Invasion

Stage IVA, according to the 2018 FIGO classification, is defined by direct invasion of cervical cancer into adjacent organs, particularly the bladder and rectum. In this context, magnetic resonance imaging (MRI) represents the most accurate imaging modality for assessing the integrity of the bladder and rectal walls, providing detailed information on pelvic anatomy and potential visceral involvement. It shows a specificity >95% and a negative predictive value (NPV) close to 100%, but with a more modest sensitivity, reported between 72–78% [20]. This means that a negative examination is highly reliable for ruling out invasion, while a positive finding must be confirmed endoscopically to avoid false positives.

The main radiological sign of visceral invasion, on T2-weighted sequences, is the interruption of the hypointense line corresponding to the muscularis propria of the bladder or rectum. This interruption may be accompanied by the presence of intramural or endoluminal nodules, but it is important to emphasize that simple contact between the tumor and adjacent viscera does not automatically imply invasion. Distinguishing between contiguity and invasion is one of the main challenges for pelvic imaging [30,31].

Diffusion-weighted imaging (DWI) sequences provide an essential contribution in characterizing equivocal areas, highlighting diffusion restriction in areas suspected of perforation or invasion, while dynamic contrast-enhanced MRI (DCE-MRI) allows assessment of the vascularization of the bladder or rectal mucosa. In the presence of invasion, early and irregular enhancement of the affected mucosal portions is observed, suggesting loss of the normal anatomical barrier between tumor and viscera [33].

Clinically, the documented presence of invasion of the bladder or rectal mucosa implies upstaging to stage IVA, with a consequent contraindication to radical surgery. Standard treatment involves the use of concomitant chemoradiation therapy (CRT). However, in case of isolated central recurrence after CRT and absence of distant metastases, pelvic exenteration may be indicated, a major surgical intervention with curative intent selected for carefully selected patients with adequate performance status [48].

PET/CT represents the gold standard for metastatic staging, thanks to its ability to identify metabolic foci even in the absence of obvious morphological alterations. The literature reviewed reports sensitivity values greater than 85% for detecting para-aortic lymph node, liver, and bone metastases, with high specificity and relatively low false-positive rates. It should be noted that PET/CT, while excellent for lymph node and metastatic assessment, lacks the same anatomical and topographical detail as MRI for direct visceral assessment. Therefore, MRI remains the preferred imaging modality for identifying and characterizing bladder and rectal invasion.

PET/MRI, an emerging technique that combines the high metabolic sensitivity of PET with the tissue resolution of MRI, has only been evaluated in preliminary studies, but offers significant translational perspectives, especially for follow-up and management of recurrences. Current data are still limited but suggest a potential diagnostic advantage in complex cases and in the presence of locoregional recurrences.

In summary, the literature agrees that mpMRI is the gold standard for assessing stage IVA, with very high specificity and an excellent negative predictive value, qualities that make it the most reliable tool for ruling out visceral invasion and guiding treatment decisions.

### 3.7. Stage IVB–Metastatic Disease

Stage IVB, according to the 2018 FIGO classification, is defined by the presence of distant metastases, which may involve extra regional lymph nodes, lungs, liver, bones, and other organs. This condition leads to a drastically worsened prognosis, with 5-year overall survival dropping to 10–20%, and requires a predominantly palliative or multimodal treatment approach, based on systemic chemoradiation, immunotherapy, or, in selected cases, salvage surgery (such as pelvic exenteration described by Höckel) [48].

In this context, conventional pelvic MRI loses its centrality, while the use of whole-body imaging techniques for systemic staging is gaining ground, including 18F-FDG PET/CT, widely recognized as the gold standard and the more recent and promising whole-body MRI with diffusion (WB-DWI/MRI).

Overall, the analysis of data broken down by FIGO stage highlights how multiparametric magnetic resonance imaging (mpMRI) is the technique of choice for the locoregional assessment of locally advanced cervical cancer, particularly in stages IIB–IIIC, thanks to its high anatomical resolution and the ability to integrate functional information. However, PET/CT shows greater sensitivity in detecting lymph node involvement and distant metastases, proving essential for advanced staging (IVA–IVB) and integrated treatment planning.

The emergence of WB-MRI as a complementary modality in advanced stages, combined with the potential of PET/MRI, suggests a future increasingly oriented toward hybrid and multiparametric approaches capable of combining diagnostic accuracy and clinical sustainability. However, the variability of protocols, the poor standardization of sequences and the heterogeneity of available studies still pose limits to the comparability and routine application of evidence, justifying the need for further prospective investigations.

## 4. Advanced Technologies and Biomarkers: Towards Precision Medicine

In recent years, the evolution of oncology imaging has extended the role of magnetic resonance imaging and hybrid techniques well beyond the simple anatomical and clinical staging of locally advanced cervical cancer (LACC). Alongside the consolidated use of PET/CT in metastatic assessment [21], emerging methodologies such as whole-body magnetic resonance imaging (WB-MRI) [47], diffusion-weighted imaging (DWI), and dynamic contrast-enhanced imaging (DCE-MRI) [27,30,31] have emerged, providing quantitative parameters that correlate with tumor biology.

These functional sequences allow the extraction of imaging biomarkers, such as the ADC (Apparent Diffusion Coefficient) and perfusion parameters (e.g., K^trans^, Fp), which have been shown to be associated with therapeutic response, tumor aggressiveness, and disease-free survival [26,27]. At the same time, artificial intelligence (AI) and radiomics have shown promising results in predicting response to chemoradiation and the risk of recurrence [15,16,18,24].

However, despite their high clinical and translational potential, these techniques are currently limited by poor standardization, differences in acquisition protocols, and the need for multicenter validation [3,4,27]. This chapter critically analyzes the main advanced technologies available today, evaluating their diagnostic performance, implementation prospects, and future role in precision medicine in gynecologic oncology.

### 4.1. PET/CT

18F-FDG PET/CT remains the reference technique for the study of metastatic disease. Numerous studies have demonstrated its superiority over conventional CT in detecting lymph node and visceral metastases, with a direct impact on therapeutic strategy. Specifically, it allows for the identification of small lung and liver metastases and the accurate definition of systemic disease spread [49].

### 4.2. Whole-Body MRI

However, in recent years, interest in whole-body MRI (WB-MRI) with DWI has grown, allowing for radiation-free systemic assessment. This approach has demonstrated increasing diagnostic utility, especially in settings where exposure to ionizing radiation is a concern, such as in young patients or during long-term follow-up. In particular, WB-DWI/MRI has proven especially effective in detecting liver and bone metastases; these are among the most common sites in advanced cervical cancers.

Liver metastases appear on MRI as hyperintense lesions on T2-weighted sequences and with diffusion restriction on DWI sequences, while bone metastases generally appear hypointense on T1-weighted sequences and hyperintense on DWI, with sensitivity often higher than CT, especially in the early detection of spinal cord lesions [50,51].

In recent years, whole-body MRI with DWI has established itself as an alternative and complementary modality. Sun et al. confirmed similar performance, highlighting the ability of WB-MRI to identify even bone lesions not always visible on PET [46]. Tanaka et al. specifically demonstrated the superiority of WB-MRI in the detection of bone metastases (sensitivity 93% vs. 76% for PET/CT), underscoring the potential of the technique in this specific setting [47]. However, it is important to emphasize that these optimal performances are not universally reproducible, as the diagnostic sensitivity of WB-MRI can vary significantly based on several factors: the acquisition protocol (magnetic fields, sequences, examination duration); the operator’s experience in image reading and the clinical setting (symptomatic patients vs. screening).

Furthermore, the lack of international standardization of protocols and interpretative criteria still limits the integration of WB-MRI into large-scale clinical workflows. For these reasons, although WB-MRI represents a promising, radiation-free alternative—particularly useful in young patients or in settings with limited access to PET/CT—it should be considered a complementary technique, not a replacement for PET/CT.

Despite the evidence in favor of WB-MRI, PET/CT currently remains the clinical standard, thanks to its greater availability and extensive multicenter validation.

However, WB-MRI offers significant advantages, such as the absence of ionizing radiation and the ability to accurately assess both visceral and bone metastases simultaneously.

### 4.3. PET/MRI

In addition to WB-MRI, a growing area of interest is PET/MRI hybrid imaging, which combines the high anatomical and tissue detail of MRI with the metabolic information provided by PET in a single acquisition, offering an integrated multiparametric assessment. This technology offers particularly promising potential in advanced staging, assessing early treatment responses, and personalized prognostic stratification, thanks to the ability to simultaneously analyze morphological, functional, and metabolic characteristics of the disease. Compared to PET/CT, PET/MRI provides better characterization of pelvic soft tissue and can reduce exposure to ionizing radiation, a significant advantage especially in young patients. Furthermore, the integration of diffusion coefficients (ADC), T2 signal intensity, and PET tracer uptake can enrich predictive models based on radiomics and artificial intelligence.

However, despite its high clinical and translational potential, PET/MRI has significant limitations to routine adoption, including its high cost, lengthy examination times, limited availability of hybrid units in clinical centers, and the need for highly specialized personnel for technical management and multiparametric data interpretation. The systematic use of PET/MRI may be justified in highly selected settings, such as clinical trials, residual disease assessments, or adaptive therapeutic strategies, but is currently limited to specialized settings. Prospective multicenter studies are desirable to evaluate its clinical efficacy in terms of survival and decision-making impact, as well as to develop standardized protocols useful for future implementation in gynecological oncology practice.

In summary, current evidence confirms that MRI remains indispensable for locoregional assessment, while PET/CT is essential for lymph node and systemic staging. New frontiers in WB-MRI could further expand diagnostic capabilities, but their application is currently limited to highly specialized centers.

### 4.4. Functional Sequences and Biomarkers

Diffusion-weighted imaging (DWI) is a fundamental component of multiparametric MRI, providing functional information on tumor cellularity. Specifically, the apparent diffusion coefficient (ADC) has shown an inverse correlation with cell density, indirectly reflecting the degree of biological aggressiveness of the tumor. Low ADC values (<1.0 × 10^−3^ mm^2^/s) have been associated with more aggressive behavior, poorer response to chemoradiation, and higher rates of local recurrence [15,52,53]. Wilson et al. (2021) demonstrated that early increases in ADC values in the first weeks of chemoradiation are associated with a greater likelihood of complete response and better local control, establishing ADC as an early predictive marker [26]. Similarly, retrospective studies have confirmed that low baseline ADC correlates with greater tumor aggressiveness and poor prognosis.

However, there are no universally accepted thresholds for risk classification, and ADC values can vary based on the acquisition protocol, magnetic field, and ROI segmentation [54]. Furthermore, although some studies have highlighted a possible association between pretreatment ADC values and disease-free survival (DFS) or overall survival (OS), multicenter histopathological validation remains limited [55,56].

DWI is particularly useful in the early assessment of treatment response, through dynamic monitoring of ADC changes during therapy [57]. However, its clinical application requires caution, as values can be influenced by necrosis, edema or technical artifacts. Therefore, integration with morphological and contrast sequences (T2 and DCE) is essential for accurate interpretation.

In addition to morphological assessment, multiparametric MRI allows the extraction of functional parameters that have become increasingly important as predictive and prognostic biomarkers.

Diffusion-weighted imaging (DWI) is one of the most studied sequences.

Perfusion imaging (DCE-MRI) provides information on tumor vascularization. Dynamic contrast-enhanced magnetic resonance imaging (DCE-MRI) provides functional information on tumor perfusion and vascular integrity by analyzing the behavior of the contrast medium within the tumor microenvironment. Among the most commonly used parameters, K^trans^ (volume transfer constant) represents the capillary transfer coefficient and reflects vascular permeability and blood flow; Higher values have been associated with more active tumor angiogenesis and, in some studies, a lower response to treatment [58]. Dickie et al. (2017) documented that parameters such as K^trans^ and plasma flow (Fp) are predictive of disease-free survival [27]. Gaustad et al. (2021) observed that elevated K^trans^ correlates with less tumor hypoxia and better clinical outcomes, strengthening the idea that DCE can identify biologically diverse subgroups and guide personalized therapies [59].

Wash-in and wash-out parameters describe the rate of contrast accumulation and elimination, respectively, and may be related to the maturity of the tumor vascular bed [58]. However, they are sensitive to technical acquisition conditions (acquisition times, contrast agent dosage and type, pharmacokinetic modeling algorithm) and require advanced software that is not always available in the clinical setting. Again, inter-center reproducibility is poor, and prospective multicenter validation is lacking. Image acquisition in DCE-MRI typically occurs with rapid T1-weighted sequences, at intervals of 5–15 s for a total period of 3–5 min, allowing for the generation of time curves and parametric maps. However, despite promising applications, standardization of acquisition and analysis protocols remains an open challenge, with marked methodological heterogeneity across studies and the absence of clinically validated thresholds for key parameters [60,61].

Therefore, although DCE-MRI represents a powerful tool in assessing tumor heterogeneity and response to therapy, its routine clinical use remains limited, and it is currently recommended as an adjunct to mpMRI, rather than as a single biomarker.

Overall, this evidence suggests that functional sequences not only increase the diagnostic accuracy of MRI but also provide quantitative biomarkers capable of predicting response and outcome, paving the way for more personalized medicine.

### 4.5. Radiomics and Artificial Intelligence

In recent years, radiomics has assumed a growing role in cervical cancer research. The extraction of quantitative features from T2-weighted images, DWI, and DCE has allowed the construction of predictive models for therapeutic response, recurrence, and lymph node metastasis.

Reinhold et al. showed that the combination of radiomic and clinical parameters significantly improves the prediction of recurrence compared to clinical data alone [16]. Liu et al. demonstrated that radiomic models derived from T2-weighted and DWI sequences achieve AUCs greater than 0.80 for the prediction of lymph node involvement, outperforming those derived from conventional radiomic models. Other authors confirmed that a low radiomics ADC correlates with poor prognosis, strengthening the relevance of quantitative biomarkers [15,29].

In parallel, studies based on artificial intelligence and deep learning have shown promising results. Jeong et al. reported accuracies > 85% in predicting complete response to chemoradiation using convolutional neural networks applied to multiparametric sequences [24]. Cai et al. demonstrated that an integrated multimodal approach (T2, DWI, and DCE) improved predictive capacity compared to models based on a single sequence [18].

Despite these encouraging results, the literature consistently highlights significant limitations: the lack of standardization of algorithms, poor reproducibility between scanners and protocols and the need for large-scale multicenter validation. For this reason, radiomics and artificial intelligence remain research tools with great potential, but not yet routinely applied in clinical practice.

To provide a quantitative summary of the diagnostic performance of the main imaging techniques, a summary table (Table 3) has been developed that collects the mean sensitivity and specificity values reported in the main studies in the literature. The data are divided by MRI sequence (T2, DWI, DCE) and by PET/CT, WB-MRI, and PET/MRI techniques, with reference to the predominant clinical application (local–regional assessment, lymph node involvement, or distant metastases).

This summary aims to assist the reader in comparing the potential and limitations of the individual techniques, facilitating clinical guidance based on stage and diagnostic objective. The values reported represent mean ranges observed, taking into account the heterogeneity between studies and the lack, in some cases, of consolidated data.

## 5. Discussion

Over the past twenty years, and particularly since 2020, MRI has established itself as a key imaging modality in the study of cervical cancer, playing a significant role in diagnosis, staging, and prognostic assessment. This systematic review confirms that multiparametric MRI is not only an imaging technique aimed at defining the morphology of the disease, but also a fundamental tool for the overall clinical management of patients with locally advanced cervical cancer (LACC).

One of the key findings is the clear superiority of MRI over traditional clinical approaches and CT in locoregional assessment. While in the past, staging was based exclusively on clinical and gynecological examination—often subject to overestimation or underestimation of parametrial and visceral invasion—today, the acquisition of high-resolution multiplanar images, with excellent soft-tissue contrast, allows for a more reliable distinction between tumors confined to the cervix and more advanced forms. It is therefore not surprising that the 2018 FIGO review formally recognized the role of imaging, and in particular MRI, in disease staging [62].

Numerous reviews and meta-analyses have demonstrated that mpMRI is the most accurate method for loco-regional assessment. In particular, Chen et al. (2024) [20] confirmed that the combination of T2-weighted sequences, DWI, and DCE significantly increases the sensitivity and specificity in diagnosing parametrial invasion compared to morphological assessment alone. These data are consistent with the conclusions of the meta-analysis by Woo et al. [19,20]. Similarly, MRI is a fundamental ally in assessing vaginal and parietal extensions, with accuracies exceeding 90% in some studies, well above those reported for clinical examination or transvaginal ultrasound. These results are consistent with the observations of Pálsdóttir et al. (2021), who documented the superiority of MRI in defining tumor margins compared to ultrasound techniques [23].

In more advanced cases, MRI plays an irreplaceable role in defining disease staging. Documentation of pelvic wall invasion or hydronephrosis is a key factor in treatment planning, marking the transition from a potentially operable disease to a condition treatable solely with chemoradiation. Recent studies have also demonstrated that hydronephrosis is not only a staging criterion but also an independent negative prognostic factor, significantly associated with reduced survival [39]. MRI, by documenting both ureteral obstruction and the locoregional disease extent, remains a cornerstone of contemporary clinical practice.

An aspect of great interest concerns the comparison between magnetic resonance imaging (MRI) and PET/CT. Although mpMRI offers excellent anatomical resolution and is essential for locoregional assessment, PET/CT provides complementary information, especially in the detection of metastatic lymph nodes and distant sites. Several studies report a sensitivity of PET/CT greater than 75–85% for lymph node involvement, compared to a sensitivity of less than 70% for mpMRI, especially in pelvic and para-aortic lymph nodes. A review of the evidence suggests that integrating the two techniques, rather than opposing them, represents the most comprehensive diagnostic approach in the setting of locally advanced cervical cancer [63,64]. However, neither technique has proven fully reliable in the diagnosis of micrometastases (<5 mm), which continue to represent a diagnostic challenge and for which surgical lymphadenectomy remains the gold standard in selected cases [36].

Recently, whole-body MRI (WB-MRI) with DWI became of interest considering it does not use ionizing radiation, represents a promising alternative, particularly relevant in young patients and in countries where PET/CT is not easily accessible. Despite the advantage of not using ionizing radiation, the technique currently has some practical limitations: prolonged acquisition times, high costs, and poor protocol standardization, which limit its clinical application to highly specialized centers. The same is true for PET/MRI, which shows excellent promise but requires advanced technological infrastructure.

Another area of growing interest concerns the evaluation of treatment response. Concomitant chemoradiotherapy (CRT) remains the standard of care in advanced stages, and MRI has proven particularly useful in monitoring tumor regression. T2-weighted sequences allow assessment of lesion volume reduction, while quantitative parameters provide the most significant data. Notably, several studies have shown that an early increase in ADC values during the first weeks of treatment is highly predictive of complete response and favorable outcome [65]. Dynamic perfusion imaging (DCE) has also shown potential prognostic value, correlating the intensity of enhancement with the degree of residual neoangiogenesis and the risk of tumor persistence (Table 4) [47].

One area in which MRI has demonstrated a significant clinical impact is the planning of MRI-guided adaptive brachytherapy (IGABT). Data from the EMBRACE studies have revolutionized clinical practice by showing that MRI-based delineation of target volumes significantly improves local control (>90%) while reducing severe toxicity (<10%) [17]. This represents a momentous shift: from two-dimensional planning based on geometric points to radiotherapy adapted to the anatomical and biological characteristics of each patient, made possible by magnetic resonance imaging. These results have also been confirmed by retrospective analyses by Sturdza and colleagues (RetroEMBRACE) and by more recent studies conducted in Asia and Europe, consolidating the idea that MRI is not only a diagnostic tool, but also an integral part of treatment [25].

These benefits translate into improved disease-free survival and quality of life, especially in intermediate- to high-risk subgroups. This evidence is recognized and integrated into the international ESGO/ESTRO/ESP guidelines (2023), which recommend the routine use of MRI for both pre-treatment assessment and high-dose brachytherapy planning.

In this context, mpMRI emerges as a cornerstone tool in the modern management of cervical cancer, with a direct and measurable impact on clinical outcomes.

One element to consider in the overall assessment of the diagnostic performance of mpMRI is the high methodological heterogeneity of the studies in the literature. Differences include the magnetic field used (1.5 T vs. 3 T), the morphological criteria employed to assess lesions, the composition of the study population (e.g., FIGO stages, histological subtypes), and whether or not functional sequences were integrated. These variables contribute to a wide variability in reported sensitivity and specificity values, with significant implications for the translation of data into clinical practice. It is therefore essential to interpret the results with caution, considering the intrinsic limitations of the existing literature and the need for prospective, multicenter studies with standardized protocols.

The future potential of multiparametric MRI, including applications of WB-MRI and artificial intelligence-based technologies, is undoubtedly promising. However, full clinical adoption of these techniques requires careful consideration of the technological and systemic barriers that remain. First, the lack of standardization in acquisition and interpretation protocols limits data comparability across centers. Second, the high cost of equipment, combined with poor accessibility in resource-limited settings, hinders equitable widespread deployment. Finally, integration into real-world clinical workflows is still limited, due to the lack of software interoperability, the shortage of trained personnel, and the absence of shared operational guidelines.

A realistic roadmap towards the clinical adoption of these technologies should include:

1. Large-scale multicenter validation of predictive models and protocols;
2. Development of international technical standards;
3. Inclusion in diagnostic-therapeutic pathways (DTPs) through pilot projects;
4. Institutional support for training and technological implementation.

Only through this integrated approach will it be possible to transform technological potential into concrete benefits for LACC management.

### 5.1. Magnetic Field

An aspect to consider concerns the magnetic field used in most of the studies analyzed: many of the sensitivity and specificity data for lymph node assessment derive from examinations performed with 1.5 Tesla MRI machines. Although this configuration is still widely used in clinical practice, the increasing adoption of 3 Tesla scanners offers the advantage of a higher signal-to-noise ratio, potentially improving the visualization of small lymph nodes and margin definition. However, the number of studies conducted with this technology is still limited, and further data are needed to confirm its actual impact on diagnostic performance in LACC.

An often overlooked but increasingly relevant aspect from a global public health perspective concerns the disparities in access to advanced imaging technologies, particularly mpMRI and PET/CT, in low- and middle-income countries (LMICs). In many of these settings, the availability of high-quality MRI scanners is limited, and access to PET/CT is often restricted to a few metropolitan centers, making it difficult to adopt optimal diagnostic protocols in the management of locally advanced cervical cancer. Added to this are challenges related to the lack of trained personnel, equipment maintenance costs, and the sustainability of radiological follow-up.

In such settings, more accessible and sustainable methods, such as transvaginal or transabdominal ultrasound, could still play a triage role, especially for assessing tumor volume, parametrial invasion, and treatment response. Although highly operator-dependent and lacking the lymph node and remote assessment capabilities of mpMRI and PET/CT, ultrasound can be a useful first step, provided it is performed by experienced operators and in integrated settings. Some studies have shown that ultrasound can achieve good accuracy in the local assessment of cervical cancer, especially in centers with gynecological expertise.

This underscores the need, even in gynecological oncology, to adapt imaging strategies to the available healthcare context, promoting the use of alternative tools where access to the most advanced technologies is limited, and encouraging training and teleradiology programs to reduce the diagnostic gap globally.

### 5.2. Radiomics and IA

The advent of radiomics and artificial intelligence opens up unprecedented scenarios. The first applications have shown how intratumoral heterogeneity, radiomic parameters and deep learning-based predictive models can estimate the risk of recurrence, the presence of lymph node metastases, and the likelihood of response to CRT. Although the application of radiomics and artificial intelligence to multiparametric MRI represents a fascinating prospect, the clinical integration of these tools remains limited by numerous technical and methodological challenges. Among the main obstacles are the lack of standardization of acquisition protocols and feature extraction methodologies, which significantly impact the reproducibility of predictive models. Furthermore, most published studies are retrospective, single-center, and based on small samples, factors that limit their external validation and clinical applicability.

Some recent studies have proposed radiomic models to predict response to chemoradiation or to distinguish between residual tissue and post-treatment fibrosis, with encouraging results in terms of diagnostic accuracy (AUC > 0.85), but still lack prospective multicenter validation. The use of AI for automatic lesion segmentation or histological classification has also been tested experimentally, but true integration into clinical workflows is currently lacking.

In the near future, the development of multi-institutional datasets, the adoption of open-source algorithms, and the use of shared frameworks for performance evaluation will be essential steps to facilitate the translation of these technologies into daily oncology practice.

In particular, their clinical implementation is still limited by technical and methodological challenges.

A typical radiomics workflow involves:

1. Manual or automatic ROI segmentation;
2. Extraction of hundreds of quantitative features (texture, shape, intensity);
3. Dimensionality reduction using feature selection algorithms (e.g., LASSO, MRMR);
4. Classification using machine learning models (e.g., support vector machine, random forest) or deep learning (e.g., CNN).

However, the heterogeneity of acquisition protocols, the lack of standardization, and the risk of overfitting represent significant obstacles to reproducibility. Harmonization techniques, such as ComBat, have been introduced to mitigate inter-scanner differences, but require further validation. Initiatives such as the Image Biomarker Standardization Initiative (IBSI) are attempting to standardize the definitions and calculation methods of radiomic features [66].

Some preliminary studies have demonstrated the ability of radiomics based on DWI or T2-weighted MRI to predict response to chemoradiation or occult lymph node involvement, with promising performance (AUC 0.75–0.89) [67,68]. However, most of these studies were conducted at single centers, with models built and validated on small and unbalanced samples, limiting their generalizability.

Therefore, while radiomics and AI have clinical potential, their use in LACC should still be considered experimental and require further multicenter, standardized, and translational studies.

In summary, in a real world clinical setting, contemporary management of LACC requires an integrated approach. The mpMRI must be strategically integrated with PET/CT and, when available, WB-MRI, within a multidisciplinary diagnostic pathway. The choice of imaging modality should be tailored to the stage of the disease, local availability, and the expected clinical decision (e.g., initial staging, therapeutic response, follow-up). An integrated, rather than sector-specific, view of the modalities is essential to optimize the management of LACC.

However, significant challenges remain: ensuring equitable access to technologies, standardizing protocols, and consolidating quantitative biomarkers and artificial intelligence models. Future prospects, linked to radiomics, artificial intelligence and the spread of whole-body MRI, outline a scenario in which imaging will no longer be simply a staging tool, but a predictive and decision-making tool at the heart of precision medicine.

Only through prospective multicenter studies and international collaborations will it be possible to transform these potentialities into concrete tools for precision medicine.

## 6. Conclusions

Multiparametric MRI remains the reference imaging modality for locoregional staging in the study of locally advanced cervical cancer: it complements PET/CT in staging, provides prognostic biomarkers, and opens new perspectives with radiomics and artificial intelligence. In clinical practice, combining MRI and PET/CT allows a comprehensive assessment of both local tumor extension and nodal or distant metastases, supporting personalized treatment planning and adaptive therapeutic strategies.

## Figures and Tables

**Table 1:** Comparison of the main mpMRI sequences in locally advanced cervical cancer: technical parameters, clinical advantages and limitations.

Sequence	Main Technical Parameters	Clinical Advantages	Limits and Critical Issues	Prognostic/Predictive Role
T2-weighted	FSE/SSFSE, thickness 3–5 mm, multiplanar planes (axial, sagittal, coronal oblique)	High anatomical resolution; definition of tumor margins; evaluation of parametrial, vaginal, and cervical invasion	Limited sensitivity in case of small tumors or minimal stromal infiltration	Indirect: useful for defining HR-CTV and residual volume
DWI	b-values ≥ 800 s/mm^2^, EPI sequences, qualitative and quantitative evaluation (ADC maps)	Early detection of CRT response; differentiates viable from necrotic tissue; supports IGABT planning	Variability between scanners and protocols; artifactual distortions; lack of standard ADC cutoffs	Yes: Pre- and post-CRT ADC correlated with response and survival
DCE-MRI	Dynamic acquisition after gadolinium; temporal resolution 5–15 s; Ktrans, Ve assessment	Tumor vascularization analysis: a potential tool for therapeutic stratification	Complex technique; poor standardization; requires dedicated software	Predictive potential (high Ktrans associated with greater response)

**Table 2:** Stratification of the strengths and weaknesses of each imagining modality by FIGO stage.

FIGO Stage	Imaging Modality	Strengths	Weaknesses
IB3–Bulky Tumors (>4 cm)	MRI	Accurate tumor volumetry; high soft-tissue resolution; essential for brachytherapy planning	Operator-dependent; may miss microscopic parametrial invasion
IIB–Parametrial Invasion	mpMRI	High specificity and NPV; T2 + DWI + DCE improves diagnostic performance; guides CRT vs. surgery	Modest sensitivity for small infiltrations; interobserver variability; requires standardized protocols
IIIA–Lower Third of Vagina	mpMRI	Superior to clinical exam and ultrasound; precise measurement of length and volume; aids brachytherapy planning	Motion artifacts or inflammation may reduce accuracy; functional sequences still under validation
IIIB–Pelvic Wall/Hydronephrosis	mpMRI	Accurate detection of pelvic wall invasion and hydronephrosis; guides CRT and urological interventions	Difficulties in bulky tumors; operator experience influences accuracy
IIIC–Lymph Node Involvement	MRI	Defines pelvic anatomy and topography; assists in radiotherapy planning	Morphological MRI criteria have low sensitivity (45–60%) for nodal metastases
	PET/CT	High sensitivity (74–100%) and specificity for nodal metastases; metabolic detection of normal-sized nodes	Limited anatomical detail for treatment planning; availability may be limited
	WB-MRI (DWI)	Radiation-free systemic assessment; good detection of bone and visceral metastases; complementary to PET/CT	Limited multicenter data; currently available only in specialized centers
IVA–Bladder/Rectal Invasion	MRI	High specificity (>95%) and NPV; detailed anatomical and topographical assessment	Sensitivity moderate (72–78%); positive findings may require endoscopic confirmation
IVB–Distant Metastases	PET/CT	Gold standard for systemic staging; high sensitivity for extra-regional metastases	Limited soft-tissue resolution; radiation exposure
	WB-MRI (DWI)	Radiation-free alternative; potential for comprehensive systemic assessment	Limited availability; requires standardization; mostly in specialized centers

**Table 3:** Sensitivity and specificity of the main imaging sequences and techniques in LACC.

Technique	Clinical Application	Sensibility	Specificity
T2-weighted MRI	Parametrial infiltration	82–89%	87–93%
DWI-MRI	Local extension, tumor margins	78–85%	80–88%
DCE-MRI	Evaluation of response to treatment	75–90%	85–90%
PET/CT	Lymph node involvement	75–88%	85–95%
PET/CT	Distant metastasis	85–90%	90–96%
WB-MRI (with DWI)	Bone and liver metastases	78–85%	87–92%
PET/MRI	Relapses and advanced stages	80–88% *	88–94% *

Note: The values reported represent mean ranges observed in the literature and may vary based on the study design, population included, and technology used. * Data derived from limited studies.

**Table 4:** The role of the main imaging techniques in LACC: from diagnosis to therapeutic impact.

Imaging Technique	Information Obtained	Direct Clinical Implication
T2-weighted MRI	Locoregional extension; parametrial invasion and adjacent structures	FIGO staging; CTV definition for IGABT
DWI/ADC mapping	Tumor viability, cellularity, response to CRT	Early assessment of response; prediction of prognosis; possible indication for surgery
DCE-MRI/Ktrans	Tumor vascular permeability; neoangiogenesis	Risk stratification; possible predictive role of response to CRT
PET/CT	Lymph node involvement and distant metastasis	Extension of the radiotherapy field; identification of target lymph nodes
WB-MRI + DWI	Evaluation of bone and liver metastases; total body staging without radiation	Alternative in young patients or in the absence of PET/CT; useful for initial staging
PET/MRI hybrid	Combined anatomical + functional information	Potential for integrated planning; still in the experimental phase
Radiomics/IA	Automated response and risk prediction using large-scale models	Currently experimental; possible future support for therapeutic decision-making

## Data Availability

No new data were created or analyzed in this study. Data sharing is not applicable to this article.

## Supplementary Materials

The following supporting information can be downloaded at: https://www.mdpi.com/article/10.3390/diagnostics15222858/s1, Table S1: Classification of the 64 studies.; Table S2: Main methodological biases in the main studies cited.
