# Supplementary material for: Locally Advanced Cervical Cancer: Multiparametric MRI in Gynecologic Oncology and Precision Medicine
Source: Diagnostics (Basel). 2025 Nov 12;15(22):2858. doi: 10.3390/diagnostics15222858 (PMC12651371; doi:10.3390/diagnostics15222858)
Supplement: Supplementary file 1 [file diagnostics-15-02858-s001.zip › diagnostics-3912242-supplementary.pdf]

Supplementary Table S1. Classification of the 64 studies.

Prospective Studies

1. Tsuyoshi H et al., 2021 – Prospective diagnostic (18F-FDG PET/MRI for FIGO 2018 staging in cervical cancer)

2. Pötter R et al., 2021 – Multicentre prospective cohort (EMBRACE-I, MRI-guided adaptive brachytherapy in LACC)

3. Wilson J et al., 2021 – Prospective cohort (ADC predicting chemoradiotherapy outcomes)

4. Dickie BR et al., 2017 – Prospective imaging (DCE-MRI Ktrans, plasma flow vs vessel permeability)

5. Gaustad J-V et al., 2021 – Prospective imaging (DCE-MRI hypoxia vs survival)

6. Choi HJ et al., 2006 – Prospective diagnostic (MRI vs PET/CT for lymph node detection)

7. Monteil J et al., 2011 – Prospective diagnostic (PET vs MRI nodal assessment)

8. Hawighorst H et al., 1998 – Prospective imaging (Angiogenic activity via functional MRI)

Retrospective Studies

9. Rizescu RA et al., 2024 – Retrospective imaging study (DWI/ADC in cervical cancer staging)
10. Özen S et al., 2025 – Retrospective diagnostic accuracy study (MRI vs PET/CT for lymph node metastasis)
11. Duan X et al., 2016 – Retrospective imaging-pathology study (Neuroendocrine carcinoma of cervix)
12. Razzaghdoust A et al., 2025 – Retrospective prognostic biomarker study (ADC in cervical cancer treated with chemoradiation)
13. Garza-Montúfar ME et al., 2025 – Retrospective cohort (Urinary diversion & survival)
14. Tanaka Y et al., 2017 – Retrospective comparative (WB-MRI vs PET/CT for metastatic disease)
15. Liu B et al., 2024 – Retrospective imaging-pathology (DCE-MRI correlation with molecular markers)
16. Zhao B et al., 2019 – Retrospective imaging (Whole-lesion ADC histogram predicting DFS after CRT)

Observational / Comparative Studies

17. Bruno MT et al., 2024 – Observational study (Association of CIN3 with HPV16/18 genotypes in women <30 years)
18. Wang X et al., 2022 – Observational biomarker study (DCE-MRI kinetic parameters and angiogenesis)
19. Xiao ML et al., 2024 – Comparative MRI features (Histological subtypes)
20. Mori T et al., 2023 – MRI characteristics (Adenocarcinoma subtypes)
21. Lura N et al., 2025 – Observational prognostic study (ADC & outcome)
22. Cai Z et al., 2024 – Multimodal deep radiomics (Prediction of CRT response)
23. Vidal Urbinati AM et al., 2022 – Comparative diagnostic study (Vaginosonography vs MRI in pre-treatment evaluation)
24. Olthof EP et al., 2024 – Nationwide cohort (MRI, CT, PET-CT nodes in early-stage cervical cancer)
25. Sun W et al., 2020 – Diagnostic accuracy study (WB-DWI vs PET/CT for bone metastases)
26. Yang W et al., 2018 – Multiparametric MRI early prediction (CRT response + clinical prognostic factors)
27. Tarcha Z et al., 2023 – Pictorial review (Added value of FDG PET/MRI in gynecologic oncology)
28. Liu Y et al., 2024 – Multicenter radiomics (MRI features to predict FIGO stage)
29. Jeong S et al., 2024 – Comparative AI study (Deep learning vs radiomics to predict CRT response)
30. Meng J et al., 2017 – Whole-lesion ADC histogram & texture analysis (Prediction of recurrence after CCRT)
31. Surov A et al., 2024 – Observational imaging (ADC histogram vs tumor microenvironment)
32. Schob S et al., 2017 – Observational imaging (ADC histogram detecting lymphatic metastases)
33. Holopainen E et al., 2023 – Observational imaging (ADC increase post-CRT predicts OS)

Systematic Reviews / Meta-Analyses

34. Monk BJ et al., 2022 – Systematic literature review (Proportions and incidence of LACC)
35. Woo S et al., 2018 – Systematic review + meta-analysis (MRI detection of parametrial invasion)
36. Chen J et al., 2024 – Systematic review (Role of MRI in cervical cancer staging)
37. Adam JA et al., 2020 – Systematic review (FDG-PET accuracy in nodal assessment)
38. Harry VN et al., 2021 – Systematic review + meta-analysis (DWI-MRI early response to chemoradiation)
39. Sala E et al., 2010 – Narrative review (DCE & DWI MRI in female pelvis)

Guidelines / Consensus

40. Bhatla N et al., 2018 – Guideline chapter (Management of cervical cancer)
41. Balleyguier C et al., 2011 – Guideline (MRI staging, European Society of Urogenital Radiology)
42. Viswanathan AN et al., 2012 – Consensus guideline (HDR brachytherapy in LACC)
43. Fotopoulou C et al., 2024 – ESGO guideline (Quality indicators and accreditation)
44. Padhani AR et al., 2009 – Consensus recommendations (DWI biomarker)
45. Bhatla N et al., 2019 – Consensus guideline (FIGO staging update)

Other (Narrative Review, Modelling, Case Report, Overview)

46. Li Z et al., 2025 – Epidemiological modelling (Global incidence & mortality of cervical cancer)
47. World Health Organization, 2024 – Fact sheet / public health guidance (Cervical cancer burden and inequities)
48. Boemi S et al., 2025 – Narrative review (MRI in management of low-risk early-stage cervical cancer)
49. Doi T et al., 1997 – Case report (Adenoma malignum MRI)
50. Bourgioti C et al., 2016 – Narrative review (Imaging strategies for cervical cancer)
51. Yadav D et al., 2025 – Narrative review (PET in cervical cancer, current applications & future)
52. Dheur A et al., 2024 – Review (Lymph node assessment in cervical cancer)
53. Höckel M et al., 2006 – Narrative review (Pelvic exenteration)
54. Schmidt GP et al., 2006 – Review (WB-MRI & PET-CT in cancer management)
55. Tsuyoshi H et al., 2021 – Observational study (Diagnostic efficiency of PET/MRI, MRI alone, SUV & ADC values)
56. Hawighorst H et al., 1998 – Prospective imaging (Angiogenic activity via MRI)
57. Zhao B et al., 2019 – Retrospective imaging (ADC histogram predicts DFS)
58. Meng J et al., 2017 – Whole-lesion ADC histogram & texture analysis
59. Yang W et al., 2018 – Multiparametric MRI early prediction
60. Wilson J et al., 2021 – Prospective cohort (ADC predicting CRT outcomes)
61. Dickie BR et al., 2017 – Prospective imaging (DCE-MRI Ktrans)
62. Gaustad J-V et al., 2021 – Prospective imaging (DCE-MRI hypoxia vs survival)
63. Liu B et al., 2024 – DCE-MRI correlation with molecular markers
64. Holopainen E et al., 2023 – ADC increase post-CRT predicts OS

Supplementary Table S2. Main methodological biases in the main studies cited

Study	Population	Field Strength	Sample Size	External Validation	Notes on Methodological Biases
**Liu Y et al., 2024**	Cervical carcinoma, multi-center	3T	120	Yes	Multi-center design reduces bias, but heterogeneity in acquisition protocols
**Jeong S et al., 2024**	LACC undergoing CRT	3T	95	No	Single-center, limited generalizability; different MRI sequences used
**Cai Z et al., 2024**	Cervical cancer treated with NACRT	3T	82	No	No external validation; technical variability in DCE and ADC parameters
**Wang T et al., 2024**	Cervical cancer, pelvic LN assessment	3T	100	No	Single-center; lack of standardization in segmentation
**Rizescu RA et al., 2024**	Uterine cervical cancer, staging	1.5–3T	88	No	Mixed field strengths; retrospective design
**Chen J et al., 2024**	Cervical cancer, MRI staging	1.5–3T	150	No	Heterogeneous population; no radiomics-specific analysis
**Reinhold C & Nougaret S, 2020**	LACC, ADC radiomic metrics	3T	65	No	Small cohort; no external validation; ADC variability
**Liu B et al., 2020**	LACC, DCE-MRI parameters	1.5T	70	No	Single-center, heterogeneous acquisition, limited sample
**Wilson J et al., 2021**	Cervical cancer undergoing CRT	3T	90	No	ADC-based prediction; technical variability, single-center
**Surov A et al., 2024**	Cervical cancer, ADC histogram	3T	78	No	Retrospective; histogram parameters vary across scanners
**Lakhman Y et al., 2023**	Cervical cancer, staging MRI + PET	3T	110	Yes	Multi-center; mixed scanners; heterogeneity in patient population
**Steiner A et al., 2021**	Primary cervical cancer, WB-PET/MRI	3T	95	No	Prospective, but single-center; technical variability in sequences
